# Evaluating decontamination protocols for the isolation of *Mycobacterium ulcerans* from swabs

**DOI:** 10.1186/s12866-016-0918-x

**Published:** 2017-01-05

**Authors:** Enid Owusu, Mercy J. Newman, Amos Akumwena, Elizabeth Bannerman, Gerd Pluschke

**Affiliations:** 1 Department of Medical Laboratory Sciences, School of Biomedical and Allied Health Sciences, University of Ghana, Accra, Ghana; 2 Department of Medical Microbiology, School of Biomedical and Allied Health Sciences, University of Ghana, Accra, Ghana; 3 Swiss Tropical Institute, Basel, Switzerland

**Keywords:** Ghana, Buruli ulcer, *Mycobacterium ulcerans*, Decontamination, Cetyl pyridinium chloride, Oxalic acid

## Abstract

**Background:**

*Mycobacterium ulcerans* (*M. ulcerans*) is the causative agent of Buruli Ulcer (BU) disease. In order to inhibit the growth of the microbial contaminants during culture of *M. ulcerans,* it is necessary to decontaminate BU samples with effective chemical agents. This study aimed at investigating some selected chemicals as potential decontamination agents for the isolation of *M. ulcerans* from swabs.

**Results:**

Povidone iodine at 0.5 and 1% exhibited the lowest contamination and recovery rate for microbial contaminants and *M. ulcerans.* The most effective decontamination method was the protocol using 2% cetylpyridinium chloride/4% sodium chloride (recovery rate = 53%, contamination rate = 14%). The observed difference between the recovery rate of 2% CPC/4% NaC and the other protocols was however not statistically significant (*p* = 0.76).

**Conclusions:**

Two percent (2%) cetylpyridinium chloride/4% sodium chloride can be conveniently used as an alternative decontamination method for the isolation of *M. ulcerans* from swabs.

## Background

*Mycobacterium ulcerans* (*M. ulcerans)* causes Buruli ulcer (BU) disease, the third most common Mycobacteriosis after tuberculosis and leprosy [1]. The disease has been reported in over 30 countries worldwide [2]. In Ghana, most endemic areas are located in water related rural farming communities, with limited access to healthcare. The associated deformities of patients as a result of the disease, coupled with the socio-economic effect on members within the affected communities makes it a public health issue [3]. Women and children under the age of 15 years are particularly affected [4]. Buruli ulcer disease manifests as skin nodules, papules, plaques, edema and ulcers. Currently, the BU disease is treated with rifampicin and streptomycin for an 8 week period [5]. Prior to the administration of the drugs however, the World Health Organization (WHO) strongly recommends that, suspected cases be confirmed by microbiological methods, including microscopy, culture, polymerase chain reaction (PCR) and histopathological analysis of diagnostic specimens from BU lesions [6].

Significant successes have been achieved with the management of BU disease since it became a global health challenge. This is as a result of intensive and extensive solution seeking investigations into a hitherto difficult situation. Investigations include (i) the extraction and use of mycolactone, the key virulent factor mediating Buruli ulcer disease for further research [7, 8], (ii) availability of information on drug susceptibility profiles of existing isolates to current antibiotics and potentially effective ones [9], (iii) *M. ulcerans’* viability in BU lesions post antibiotic treatment, indicative of treatment successes or failures, (iv) molecular epidemiology of the disease [10] and (v) disease surveillance. Most of these investigations have relied on *M. ulcerans* isolates from culture; a method for growing isolates of viable *M. ulcerans* in-vitro*.* This process therefore remains a very vital and important tool, for effective disease management. The culture process requires the use of diagnostic specimens from BU lesions. Faster growing microbial contaminants in BU lesions overgrow the *M.ulcerans* cultures, affecting their yield [11]. The reduction of these contaminants is usually achieved by processing the samples with chemical agents having selective antimicrobial properties in a process referred to as decontamination [12]. Effective decontamination protocols generally inhibit the growth of microbial contaminants with little or no inhibitory effect on viable *M. ulcerans* [13].

Commonly employed decontamination procedures in the primary isolation of *M. ulcerans* include sodium chloride (Petroff method) [14, 15], oxalic acid [14], N-acetyl cysteine [15, 16], Sulfuric Acid [16] (Buijtels PC1, Petit PL), benzalkonium chloride (BC) [17], trisodium Phosphate [15], sodium lauryl sulfate [18], and the equivalent 1- Hexadecylpyridinium chloride (HPC) methods [19–21]. To date, almost all decontamination protocols have their associated challenges [22, 23]. These include higher toxicity to *M. ulceren* and slow processing time due to the reason that most protocols require a neutralization stage [16, 22].

Diagnostic specimens commonly used for *M. ulcerans* isolation include tissue samples, swabs and fine needle aspirates [10, 24]. Recovery from tissue specimens processed with 5% oxalic acid remains the highest reported decontamination process so far at 78% [25]. The effective use of antibiotics as definitive BU treatment has limited the availability of tissue samples [26], making swabs and fine needle aspirates more convenient alternatives. Decontamination protocols used for swabs should be relatively less harsh to bacilli in the already paucibacillary specimen. In addition, large open BU lesions tend to harbour spores of microbes including fungi and other hardy environmental microorganisms. To address these challenges, there is the need to design more decontamination protocols, not only with the sole objective of inhibiting growth of vegetative bacteria but also fungal contaminants [27]. Effective decontamination methods (especially chemical agents with known antifungal properties) can complement the commonly used 5% oxalic acid. The current study therefore investigated some chemicals as decontamination agents for the isolation of *M. ulcerans* from swabs.

## Methods

Investigations and sample processing took place at the Bacteriology Department of the Noguchi Memorial Institute for Medical Research (NMIMR), University of Ghana, Legon, Tuberculosis Laboratory of the Public Health Reference Laboratory (PHRL), of the Ministry of Health and the chest clinic of the Korle-Bu Teaching Hospital (KBTH), Korle-Bu. This research was carried out between the periods June 2010 and April 2012.

### Preparation of Buruli ulcer samples and clinical isolates

Exudates from BU lesions of 35 subjects were swabbed for the study. Subjects were clinically suspected BU cases who reported at the Paakro and Asuboi health centers of the Eastern region of Ghana. The selected samples had been authenticated as being *M. ulcerans* positive by microscopy and IS*2404* polymerase chain reaction (PCR). Specimen collection process involved a circular manipulation of the swab within the periphery of lesions [27].

Swabs in their receptacles were kept under cold storage and transported to the laboratory for analysis. Each swab specimen was eluted in 2 ml (2 ml) of sterile phosphate buffered saline (PBS) in a 15 ml capacity screw-capped test tube (3 mm diameter, Merck, Germany) [25].

Buruli ulcer sample for analysis was obtained as an eluted suspension, facilitated by vortexing swab tips with 10–15 glass beads for a minute. Bacterial clinical isolates used in the study included *Pseudomonas aeruginosa, Salmonella typhi, Escherichia coli, Bacillus cereus, Staphylococcus epidermidis, Staphylococcus aureus* and *Klebsiella pneumonia. Aspergillus niger* and *Candida albicans* were the only fungi isolates tested. Each microbial inoculum was prepared from a loopful of pure microbial culture emulsified in sterile distilled water to obtain turbidity comparable to McFarland’s standard solution 0.5, equivalents to 1×10^6^ bacteria cells/ml.

### Chemical agents preparation

Five (5) selected chemical agents, prepared at indicated working dilutions were used. These were (i) 0.5% povidone iodine (PI) [28], (ii) 1% povidone iodine (PI), (iii) 1% cetylpyridinium chloride/2% sodium chloride (iv) 2% cetyl-pyridinium chloride/4% sodium chloride, (v) 5% oxalic acid (vi) 10% oxalic acid [29] (vii) 0.5% virkon and (viii) 1% virkon (ix) 20% benzalkonium chloride and (x) benzalkonium chloride at 40% [30, 31]. Therefore, two dilutions each of the 5 chemical agents were prepared and used. Hence, 10 combinations were used for the subsequent process. Sterile distilled water was used as diluents for all chemical agents.

### Validating selected chemical agents as effective antimicrobials

Each of the five chemical agents prepared in the dilutions indicated was mixed by vortexing with suspensions of microbial inoculums of the potential contaminants (*P. aeruginosa, S. typhi, E. coli, B. cereus, S. epidermidis, S. aureus, K. pneumonia*, *A. niger* and *C. albicans*) in equal volumes of 200 μl per test. Inoculums and chemical agents were vortexed for a minute and incubated at room temperature for 15 min with intermittent mixing. This process was aimed at ensuring even distribution and exposure. One Hundred microliters (100 μl) of each test was inoculated onto Mueller-Hinton (MH) agar plates and evenly spread to ensure uniformity after which the plates were incubated at 37 °C for 24 h. A test showing no growth was indicative of an effective inhibitory effect of the tested chemical agents at the prepared dilution.

### Inhibitory effect of chemical agents on microbial contaminants in BU samples

Benzalkonium chloride was the only chemical agent that did not go through the validation stage successfully. Therefore, four successfully validated chemical agents were subsequently tested for their antimicrobial activity against microbial contaminants in BU samples. This implies that the number of chemical agents used for the study became 4 and in two dilutions each, making it 8 combinations. In brief, samples were obtained as swabbed exudates and pus from the periphery and crevices of the ulcers. Sample suspensions were obtained by agitating swab tips in fixed volume (2 ml) of sterile phosphate buffered saline and glass beads. The suspension was then aseptically transferred to another sterile tube. For each of the suspensions an adjusted turbidity comparable to MacFarlands standard solution 1 was used. Chemical agents were mixed with BU sample suspensions in equal volumes of 400 μl. Tests were vortexed to ensure adequate exposure, and incubated at room temperature for 15 min. One hundred microliters (100 μl) of well mixed test was inoculated on Mueller Hinton agar plates and incubated at 37 °C for 24 h. Media plates showing no growth was indicative of the antimicrobial effect of the chemical agent on the microbial contaminants in the BU samples.

### Examination of chemicals as effective decontamination agents

The selected chemical agents at the specified dilutions were investigated for their potential as suitable decontamination agents for the recovery of *M. ulcerans* from swab specimens [32].

Two milliliters (2 ml) aliquots of the sample homogenates were subjected to eight decontamination protocols using four chemical agents. The protocols were cetylpyridinium chloride at concentrations of 0.5 and 1%, povidone iodine antiseptic at concentrations of 0.5 and 1%, oxalic acid at concentrations of 5 and 10%, and virkon disinfectant at concentrations of 0.5 and 1%. Briefly, each of the test samples was incubated for 30 min under room temperature conditions after which they were centrifuged at 3000 rpm for 15 min. The supernatant was discarded and the pellet obtained re-suspended in 1.0 mL of sterile phosphate buffered saline (PBS) solution to obtain sample inoculums. Each sample inoculum (100 μL) was seeded on Lowenstein-Jensen (L-J) slants in duplicates and gently spread over the surface. The tubes were loosely covered and examined daily until the media surface was completely dried. They were then tightly capped, arranged at a slanted angle of 30° and incubated at 32 °C. The cultures were examined weekly for the growth of contaminants and *M. ulcerans* (slow-growers) for 12 weeks. The *M. ulcerans* colonies were identified by conventional laboratory methods [33] by subjecting the cultures with growth to Ziehl-Neelsen staining and a confirmatory IS2404 PCR [33]. Cultures showing no *M. ulcerans* growth after 12 weeks were considered negative, whilst those with growth were considered positive. Contaminated cultures were those exhibiting over 50% growth coverage of colonies other than that of *M. ulcerans*. Chemicals assessed as effective decontamination agents were the tests that exhibited high *M. ulcerans* recovery and low contamination rates. Controls for media, chemical agents and bacteria were set alongside all three experimental processes; testing the selected chemical agents (on test organisms) as effective antimicrobials, examination for their antimicrobial activity against microbial contaminants in BU samples and their potential as suitable decontamination agents for the recovery of *M. ulcerans* from swab specimens. They were incubated and read simultaneously with the tests at 32 °C [34].

### Data analyses

Data from this study was stored in Microsoft excel (MS Excel) and analyzed with STATA 11 (Strata Corp, College Station, TX). Data analysis involved descriptions of estimated totals, arithmetic means, ranges and prevalence rates of the study variables. Association between the protocols was determined using the chi-square (*χ*2) test, and based on 95% confidence interval. A *P-*Value less than 0.05 was considered statistically significant.

## Results

Validation of selected chemicals as effective antimicrobial agents displayed various levels of activity against the test bacterial isolates *(P. aeruginosa, S. typhi, E. coli, Bacillus cereus Staphylococcus epidermidis, Staphylococcus aureus, K. pneumoniae)* and fungal isolates (*A. niger* and *C. albicans*) (Table 1). All the test microbes were susceptible to both concentrations of povidone iodine (Table 1). *E. coli, P. aeruginosa* and *S. typhi* exhibited resistance to 1% CPC/2% NaCl whilst *Bacillus cereus* and *A. niger* exhibited resistance to 5% oxalic acid. Virkon disinfectant at 0.5% exhibited antimicrobial activity against all except *A. niger (*Table 1). Meanwhile, 20% BC (Timsen) and 40% BC (Timsen) had no antimicrobial activity against *P. aeruginosa, B. cereus* and *A. niger.*

**Table 1:** Inhibitory effect of selected chemical agents on clinical isolates of Potential skin contaminants

Chemical agents	In-vitro resistance of microbes to chemical agents^a^	Test plates showing no growth, n (*N* = 70)	Estimated activity levels of chemical agents (%)
0.5% PI	None of the microbes tested	68	97
1% PI	None of the microbes tested	70	100
1% CPC/2% NaCl	*E. coli, P. aeruginosa & S. typhi*	65	92
2% CPC/4% NaCl	None of the microbes tested	67	95
5% Oxalic acid	*B. cereus & A. niger*	64	91
10% Oxalic acid	None of the microbes tested	67	96
0.5% Virkon	*A. niger*	67	96
1% Virkon	None of the microbes tested	67	96
20% BC (Timsen)	*P. aeruginosa, B. cereus & A. niger*	60	86
40% BC (Timsen)	*P. aeruginosa & A. niger*	63	90

*PI* Povidone iodine, *CPC* Cetyl pyridinium chloride, *NaCl* Sodium chloride, *BC* Benzalkonium chloride

^a^Test microbes were *P. aeruginosa, S. typhi, E. coli, Bacillus cereus, S. epidermidis, S. aureus, K. pneumonia, A. niger* and *C. albicans*. % = n/N × 100

Various degrees of growth inhibition on the samples were observed for the thirty five (35) BU samples (processed in duplicates to get a total of 70 samples and) tested with the selected chemical agents. Levels of inhibition which shows how many of plates were inhibited out of the 70 by a particular protocol ranged between 86% (60/70) and 100% (70/70). The highest levels of inhibition was exhibited by 0.5% povidone iodine and 1% povidone iodine; 97% (68/70 plates) and 100% (70/70 plates) respectively. The activities of the chemical agents against the microbial sample contaminants were estimated based on the level of growth inhibition exhibited by test plates. The lowest level of 86% of antimicrobial activity was exhibited by 20% benzalkonium chloride (BC). Five percent oxalic acid exhibited an activity level of 91%.

A summary of the effect of the eight decontamination protocols investigated against 35 BU sample suspensions (prepared in duplicates) for the primary isolation of *M. ulcerans* from the BU samples is shown in Table 2. The recovery rates ranged between 0 and 53%. The highest *M. ulcerans’* recovery rate was by 2% CPC/4% NaCl at 53% whilst the lowest was by 0.5 and 1% povidone iodine (Table 2). The rate for 5% oxalic acid was 44%. The secondary contamination rates of the *M. ulcerans* cultures were assessed for the various protocols. The least rate of secondary contamination was observed in all the two dilutions of povidone iodine (Table 2). The highest rate (29%) of secondary contamination was recorded for the conventional (5% oxalic acid) decontamination protocol followed by 1% CPC/2% NaCl (21%) (Table 2).

**Table 2:** Effect of protocols on the recovery of *M. ulcerans* from BU samples, in-vitro

Decontamination protocols	No. of tubes with *M. ulcerans* growth per protocol^a^	Recovery rate (%) (*p*-value)	No. of tubes with contamination^a^	Contamination rate (%) (*p*-value)
0.5% PI	0	0 (0.00)	0	0 (0.00)
1% PI^b^	0	0 (0.00)	0	0 (0.00)
1% CP/2% NaCl	33	47 (0.00)	15	21 (0.00)
2% CPC/4% NaCl	37	5 3(0.76)	10	14 (0.00)
5% Oxalic acid	31	44 (0.07)	20	29 (0.00)
10% Oxalic acid	10	14 (0.00)	12	17 (0.00)
0.5% Virkon	36	51 (0.40)	12	17 (0.00)
1% Virkon	6	0.1 (0.00)	7	10 (0.00)

^a^ *n* = total number of tests: 35 × 2 = 70,

^b^Conventional method, *BU* Buruli ulcer, *PI* Povidone iodine, *CPC* Cetyl pyridinium chloridem, *NaCl* Sodium chloride

## Discussion

A major challenge in the primary isolation of *M. ulcerans is* contamination by fast growing microbes. Swab specimens, a more convenient diagnostic specimen tend to have lower yields due to limitations in sample collection. Optimization based studies aimed at complementing current decontamination protocols are essential. This study therefore investigated five chemicals for their effectiveness as decontamination agents in isolating *M. ulcerans* from swabs. Among the chemical agents investigated was the currently used 5% oxalic. The study was designed to preliminarily validate their effect on some likely bacterial and fungal skin contaminants. Among the bacterial contaminants were *Bacillus cereus, E. coli, K. pneumoniae, K. species, P. aeruginosa, S.typhi, S. epidermidis* and *S. aureus. Candida albicans* and *Aspergillus niger* were the fungal contaminants used in the validation. Microbial contaminants isolated from BU samples collected for the study were also used in the validation process.

All tested chemical agents demonstrated varying degrees of inhibition against the isolates at their prepared dilutions. The effectiveness of povidone iodine and virkon as antimicrobial agents was clearly seen; as they exhibited the highest activity at 98 whilst 20% benzalkonium chloride (an antiseptic for the cleaning of diabetic wounds) showed the lowest activity at 86%. In spite of high activity recorded by some of the chemical agents, *P. aeruginosa and A. niger* proved to be the most challenging to inhibit among all the microbial isolates tested*.* This finding brings to the fore the intrinsic resistance properties exhibited by *P. eruginosa* to most antimicrobials [11]. Similar observations were made by McClean et al. [11], where they also observed that *P. aeruginosa w*as the predominant contaminant associated with the conventional isolation of *Mycobacterium tuberculosis*. That study also reported that 5% oxalic acid alone and in combination with NaOH was effective in eliminating *P. aeruginosa* [11]. The challenge in eliminating *Aspergillus niger* with the chemical agents used in the current study could be attributed to the fact that, most vegetative fungi and fungal spores are more resistant to decontamination agent. Biocide-microbial interaction usually occurs at the surface with little impact on the fungi, as few biocides are intended to focus on fungus cell as a major target.

The highest mean recovery of *M. ulcerans* from culture was exhibited by 2% cetyl pyridinium chloride/4% sodium chloride (53.0%) and 0.5% virkon (51.0%) treated samples, and the lowest was observed in samples treated with 0.5 and 1% povidone iodine (0%). The recovery rate of 1% cetyl pyridinium chloride/2% sodium chloride and 5% oxalic acid were comparable at 44 and 41% respectively. This recovery rates are relatively less than 0.5 and 1% virkon disinfectant and 2% CPC/4% NaCl. In this study, *M. ulcerans* yield from 5% oxalic acid was comparatively lower at 41%. This observation conforms to outcome from similar study conducted by Palomino and Portaels [14], where they observed a marked reduction in the growth rate of *M. ulcerans* in culture due to the harmful effect of oxalic acid on the culture of *M. ulcerans* from the BACTEC system [14]. Other investigators have however reported on high yields with the use of 5% oxalic acid. In a study by Yajko et al. [35], where they worked on fecal samples cultured on Lowenstein-Jensen media to evaluate various chemical decontamination protocols, they reported that the highest yield of *Mycobacterium avium* complex (MAC) was with oxalic acid [35]. Another study conducted in Ghana using 5% oxalic acid gave an *M. ulcerans* isolation rate of 78% from tissue [25], and this is relatively higher compared to the 41% recovery rate observed for 5% oxalic acid from swabs.

The observed differences in the various studies including the current one appear to be due to various factors such as specimen types and treatment processes used, in addition to the array of selected chemical agents used for the evaluation studies. This study on the other hand, used swab specimens from open ulcers, cultured on Lowenstein-Jensen media. This confirms the variability of outcomes for recovery of *M. ulcerans* from culture, based on the specimens and the media used. The implication could be that whilst the decontamination step is very important for *M. ulcerans’* recovery, other factors could also affect the yield. In addition, it is possible that heavily contaminated specimens would require harsher treatments, but could also affect the viability of the target organism (*M. ulcerans)* in the sample.

Cetyl pyridinium chloride in sodium chloride though comparatively less harsh than povidone iodine, exhibited the best *M. ulcerans* recovery rate in this study. This phenomenon may be attributed to the antifungal properties of CPC/NaCl [20, 27] and its usefulness for the transport of specimens from peripheral centers to the reference laboratories for analysis, especially in under-resourced endemic areas [19]. Anti-Fungal agents control the overgrowth of fungi, which characteristically spread at a rapid rate over the media surface. This implies that decontamination protocols incorporating anti-fungal properties will be required for effective decontamination.

This study has demonstrated that, single decontamination protocol only cannot improve the recovery of *M. ulcerans* from clinical specimens, especially swabs. In the culture of slow growing *Mycobacterium*, cultures are examined on a daily basis for 7 days for the presence of faster growing microbial contaminants. Even though povidone iodine was preliminarily validated as an effective antimicrobial agent against all contaminants tested, no colonies of *M. ulcerans* were observed. This outcome could be due to the fact that povidone iodine has a sterilizing effect in culture; property that made it unsuitable as a decontamination agents at the dilutions investigated in the current study. This observation corroborates the fact that, in investigating potentially novel decontamination protocols, a stepwise approach of validating its effect as a decontaminant at the specified dilution would be required. This would help reduce errors associated with the selection of appropriate decontamination protocols based on the premise that effective antimicrobial agents are by extension effective decontamination agents for the isolation of *Mycobacterium*. In addition, optimization of decontamination protocols for the isolation of *Mycobacterium* species would require the use of lower concentrations of the test agent and longer periods of exposure.

In a different study, Smithwick et al. [19] found 1% cetyl pyridinium chloride/2% sodium chloride useful for the transport of sputum specimens from peripheral to reference laboratories. They observed that its effect was comparable to the conventionally used Nacetyl- L-cysteine. Results from this study corroborated his observation and also found it useful. Whilst the 1% povidone iodine protocol did not yield any *M. ulcerans* from culture, it recorded no contamination. This was also observed for 1% virkon disinfectant. Meanwhile, the highest rate (29%) of secondary contamination was recorded for the conventional (5% oxalic acid) protocol. Although this is a strong decontamination protocol, experimental period of exposure and low a concentration of agent in the culture medium may contribute to this contamination rate [11].

To the best of our knowledge, the use of povidone iodine and virkon as a decontamination agent in the primary isolation of *Mycobacterium ulcerans* in this study represents the first attempt to evaluate these chemical agents. The decontamination protocols utilizing the two concentrations of povidone iodine seems to affect not only the viability of the contaminating microbes, but it also inhibits the growth of the *M. ulcerans* in culture. This informs future projections where povidone iodine could be used at relatively lower concentrations to achieve more desirable results. Also, this study reports the effective use of 0.5% virkon, a broad spectrum disinfectant for the isolation of *M. ulcerans* from swabs.

## Conclusions

Effective means of isolating *M. ulcerans* from diagnostic specimens, particularly swabs is required for control of BUD. This will inform policy on effective disease control measures targeted at reducing BU cases. The current research has demonstrated that, 2% cetylpyridinium chloride/4% sodium chloride can be conveniently used as an alternative decontamination method for the isolation of *M. ulcerans* from swabs. This can contribute towards effective diagnosis and control of BU disease, worldwide.

## Abbreviations

BC: Benzalkonium chloride
BU: Buruli ulcer
BUD: Buruli ulcer disease
CPC/NaCl: Cetyl
CPC: Cetyl pyridinium chloride
GHS: Ghana Health Service
HPC: Hexa-decylpyridinium chloride
KBTH: Korle-Bu Teaching Hospital
*M. ulcerans*: *Mycobacterium ulcerans*
ml: millilitres
NaCl: Sodium chloride
NMIMR: Noguchi Memorial Institute for Medical Research
OA: Oxalic Acid
PBS: Phosphate buffered saline
PCR: Polymerase chain reaction
PI: Povidone iodine pyridinium chloride in Sodium chloride
WHO: World Health Organization

## References

[1] Walsh DS, Portaels F, Meyers WM (2010). Recent advances in leprosy and Buruli ulcer (Mycobacterium ulcerans infection). Curr Opin Infect Dis.

[2] Thangaranj HS, Evans MRW, Wansbrough-Jones MH (1999). *Mycobacterium ulcerans* disease; Buruli ulcer. Trans Royal Soc Trop Med and Hyg.

[3] Aiga H, Amano T, Cairncross S, Adomako J, Nanas OK (2004). Assessing water-related risk factors for Buruli ulcer: A case-control study in Ghana. Am J Trop Med Hyg.

[4] Asiedu K, Etuaful S (1998). Socioeconomic implications of Buruli ulcer in Ghana: a three year review. Am J Trop Med Hyg.

[5] World Health Organization (2004). Provisional guidance on the role of specific antibiotics in the management of *Mycobacterium ulcerans* disease (Buruli ulcer).

[6] World Health Organization (2001). Buruli ulcer. Diagnosis of Mycobacterium ulcerans disease. A manual for health care providers.

[7] Adusumilli S, Mve-Obiang A, Sparer T, Meyers W, Hayman J, Small PL (2005). *Mycobacterium ulcerans* toxic macrolide, mycolactone modulates the host immune response and cellular location of *M. ulcerans* in vitro and in vivo. Cell Microbiol.

[8] Ortiz RH, Aguilar L, Estevez HO, Martin A, Herrera JL, Romo LF (2009). Differences in virulence and immune response induced in murine model by isolates of *Mycobacterium ulcerans* from different geographic areas. Clin Exp Immunol.

[9] Marsollier L, Honore’ N, Legras P, Manceau AL, Kouakou H, Carbonnelle B (2003). Isolation of three *Mycobacterium ulcerans* strains resistant to rifampin after experimental chemotherapy of mice. Antimicrob Agents Chemother.

[10] Walsh DS, Eyase F, Onyango D, Odindo A, Otieno W, Waitumbi JN (2009). Clinical and molecular evidence for case of buruli ulcer (M.ulcerans) in kenya. Am J Trop Med Hyg.

[11] McClean M, Stanley T, Stanley S, Maeda Y, Goldsmith CE, Shepard R (2011). Identification and characterization of breakthrough contaminants associated with the conventional isolation of Mycobacterium tuberculosis. J Med Microbiol.

[12] Bratschi MW, Ruf M-T, Andreoli A, Minyem JC, Kerber S, Wantong FG (2014). Mycobacterium ulcerans Persistence at a Village Water Source of Buruli Ulcer Patients. PLoS Negl Trop Dis.

[13] Collins C, Grange J, Yates MD (1997). Tuberculosis bacteriology (organization and practice) *Reed Educational and professional Publishing Ltd UK*.

[14] Palomino JC, Portaels F (1998). Effects of decontamination methods and culture conditions on viability of *Mycobacterium ulcerans* in the BACTEC system. J Clin Microbiol.

[15] Chatterjee M, Bhattacharya S, Karak K, Dastidar SG (2013). Effects of different methods of decontamination for successful cultivation of Mycobacterium tuberculosis. Indian J Med Res.

[16] Buijtels PC, Petit PL (2005). Comparison of NaOH-N-acetyl cysteine and sulfuric acid decontamination methods for recovery of mycobacteria from clinical specimens. J Microbiol Methods.

[17] Zabel LT, Albus J, Eirinq P (1998). Comparison of the effect of two decontamination procedures on the detection of *Mycobacterium tuberculosis* complex in respiratory specimens by a target-amplified test. Clin Microbiol Infect.

[18] Manterola JM, Thornton CG, Padilla E, Lonca L, Corea I, Martinez E, Ausina V (2003). Comparison of the sodium dodecyl sulfate-sodium hydroxide specimen processing method with the C_18_-carboxypropylbetaine specimen processing method using the MB/BacT liquid culture system. Eur J Clin Microbiol Infect Dis.

[19] Smithwick RW, Stratigos CB, David HL (1975). Use of cetylpyridinium chloride and sodium chloride for the decontamination of sputum specimens that are transported to the laboratory for the isolation of *Mycobacterium tuberculosis*. J Clin Microbiol.

[20] Pardini M, Varaine F, Iona E, Arzumanian E, Checchi F, Oggioni MR (2005). Cetylpyridinium chloride is useful for isolation of Mycobacterium tuberculosis from sputa subjected to long-term storage. J Clin Microbiol.

[21] Corner LA, Trajstman AC (1988). An evaluation of 1- Hexadecylpyridinium Chloride as a decontaminant in the primary isolation of *Mycobacterium bovis* from bovine lesions. Vet Microbiol.

[22] Ambrosio SR, Oliveira EM, Rodriguez CAR, Neto FJS, Amaku M (2008). Comparison of three decontamination methods for *Mycobacterium bovis* isolation. Braz J Microbiol.

[23] Corner LA, Trajstman AC, Lund K (1995). Determination of optimum concentration of decontaminants for primary isolation. New Zeal Vet J.

[24] Cassisa V, Chauty A, Marion E, Ardant MF, Eyangoh S, Cottin J (2010). Use of Fine-Needle Aspiration for Diagnosis of Mycobacterium ulcerans Infection. J Clin Microbiol.

[25] Yeboah-Manu D, Bodmer T, Mensah-Quainoo E, Owusu S, Ofori-Adjei D (2004). Evaluation of decontamination methods and growth media for primary isolation of *Mycobacterium ulcerans* from surgical specimens. J Clin Microbiol.

[26] Cornet L, Richard-Kadio M, N’Guessen HA, Yapo P, Hossoko H, Dick R (1992). Treatment of Buruli ulcers by excision-graft. Bull Soc Pathol Exot.

[27] Phillips BJ, Kaplan W (1976). Effect of cetylpyridinium chloride on pathogenic fungi and Nocardia asteroides in sputum. J Clin Microbiol.

[28] Reimer K, Fleischer WA (1997). Povidone-iodine in antisepsis-State of the art. Dermatology.

[29] Riemenschneider W, Tanifuji M (2002). “Oxalic acid”. in Ullmann’s Encyclopedia of Industrial Chemistry.

[30] Gasparini R, Pozzi T, Magnelli R, Fatighenti D, Giotti E, Poliseno G (1995). Evaluation of in vitro efficacy of the disinfectant Virkon. Eur J Epidermiology.

[31] Hernandez A, Martro E, Matas L, Martin M, Ausina V (2000). Assessment of in-vitro efficacy of 1% Virkon against bacteria, fungi, viruses and spores by means of AFNOR guidelines. J Hosp Infect.

[32] Kent PT, Kubica G (1985). Public health mycobacteriology: A guide for the level III laboratory.

[33] Herbinger KH, Adjei O, Awua-Boateng NY, Nienhuis WA, Kunaa L, Siegmund V (2009). Comparative study of the sensitivity of different diagnostic methods for the laboratory diagnosis of Buruli ulcer disease. Clin Infect Dis.

[34] Grandjean L, Martin L, Gilman R, Valencia T, Herrera B, Quino W (2008). TB & MDRTB testing by direct sputum culture in selective broth without decontamination or centrifugation. J Clin Microbiol.

[35] Yajko DM, Nassos PS, Sanders CA, Gonzalez PC, Reingold AL, Horsburgh CR (1993). Comparison of four decontamination methods for recovery of Mycobacterium avium complex from stools. J Clin Microbiol.

